# Acne and Stress: Impact of Catecholamines on *Cutibacterium acnes*

**DOI:** 10.3389/fmed.2019.00155

**Published:** 2019-07-10

**Authors:** Valérie Borrel, Pauline Thomas, Chloé Catovic, Pierre-Jean Racine, Yoan Konto-Ghiorghi, Luc Lefeuvre, Cécile Duclairoir-Poc, Christos C. Zouboulis, Marc G. J. Feuilloley

**Affiliations:** ^1^Laboratory of Microbiology Signals and Microenvironment LMSM EA4312, University of Rouen Normandy, Normandie Université, Evreux, France; ^2^R&D Uriage Dermatological Laboratory, Neuilly sur Seine, France; ^3^Departments of Dermatology, Venereology, Allergology, and Immunology, Dessau Medical Center, Brandenburg Medical School Theodor Fontane, Dessau, Germany

**Keywords:** epinephrine, norepinephrine, microbial endocrinology, biofilm, bacterial surface polarity, cytotoxicity, inflammation

## Abstract

*Cutibacterium acnes* (former *Propionibacterium acnes*), is a bacterium characterized by high genomic variability, consisting of four subtypes and six major ribotypes. Skin is the largest neuroendocrine organ of the human body and many cutaneous hormones and neurohormones can modulate *b*acterial physiology. Here, we investigated the effect of catecholamines, i.e., epinephrine and norepinephrine, on two representative strains of *C. acnes*, of which the genome has been fully sequenced, identified as RT4 acneic and RT6 non-acneic strains. Epinephrine and norepinephrine (10^−6^ M) had no impact on the growth of *C. acnes* but epinephrine increased RT4 and RT6 biofilm formation, as measured by crystal violet staining, whereas norepinephrine was only active on the RT4 strain. We obtained the same results by confocal microscopy with the RT4 strain, whereas there was no effect of either catecholamine on the RT6 strain. However, this strain was also sensitive to catecholamines, as shown by MATs tests, as epinephrine and norepinephrine affected its surface polarity. Flow cytometry studies revealed that epinephrine and norepinephrine are unable to induce major changes of bacterial surface properties and membrane integrity. Exposure of sebocytes to control or catecholamine-treated bacteria showed epinephrine and norepinephrine to have no effect on the cytotoxic or inflammatory potential of either *C. acnes* strains but to stimulate their effect on sebocyte lipid synthesis. Uriage thermal spring water was previously shown to inhibit biofilm production by *C. acnes*. We thus tested its effect after exposure of the bacteria to epinephrine and norepinephrine. The effect of the thermal water on the response of *C. acnes* to catecholamines depended on the surface on which the biofilm was grown. Finally, an *in-silico* study revealed the presence of a protein in the genome of *C. acnes* that shows homology with the catecholamine receptor of *Escherichia coli* and eukaryotes. This study suggests that *C. acnes* may play a role as a relay between stress mediators (catecholamines) and acne.

## Introduction

Catecholamines (epinephrine, norepinephrine, and dopamine) are small cyclic compounds derived from tyrosine. Known to be neurotransmitters and hormones in vertebrates, they can also act on bacteria. This observation was the origin of the concept of “Microbial endocrinology,” i.e., the detection of human endocrine or neuroendocrine factors by microorganisms (1). The roots of this concept come from the demonstration that the administration of epinephrine to patients can induce a burst of preexisting bacterial infections (2). However, it was necessary to wait for the studies of Lyte and Ernst (3), and later Sperandio et al. (4), before the scientific community accepted that bacteria actually express specific catecholamine receptors. The first studies were performed using *Escherichia coli* as a model, but it has now been demonstrated that many Gram-negative and Gram-positive bacteria can respond to catecholamines (5, 6). The concept of microbial endocrinology was later broadened to a large variety of eukaryotic communication molecules, ranging from cytokines to neuropeptides (7).

The interaction of catecholamines with bacteria has been particularly investigated in the gastro-intestinal tract, where it was demonstrated that epinephrine and norepinephrine play an important role in the stimulation of enteric pathogens, such as enterohemorrhagic *E. coli* (8). It was also observed that the sympathetic neurotransmitter norepinephrine promotes expression of PA-1 lectin/adhesin in *Pseudomonas aeruginosa*, resulting in an increase in adhesion to colonocytes and the alteration of trans-epithelial permeability (9). *Pseudomonas fluorescens*, a closely related species, responds similarly to epinephrine by destabilizing the intestinal epithelial barrier (10). Thus, the sympathetic network of the gastrointestinal tract appears to be an essential element of the gut microbiota-brain axis (11).

Skin harbors the second largest microbiota of the human body after the gut (12). Overall, 25–50% of sympathetic nerve terminals target skin effectors, making catecholamines the principal autonomic skin neurotransmitters (13, 14). Moreover, keratinocytes and melanocytes themselves have the capacity to synthesize and metabolize catecholamines (15). Skin thus may be a target, as well as an active element, in the hypothalamic-pituitary-adrenocortical axis activated during stress (16). It has been recently demonstrated that cutaneous bacteria, including *Staphylococcus aureus, Staphylococcus epidermidis, Pseudomonas fluorescens, Bacillus cereus*, and *Cutibacterium* (former *Propionibacterium*) *acnes* can sense and react to skin neuropeptides, such as substance P, calcitonin gene related peptide, and natriuretic peptides (17–20). Thus, the skin microbiota may be a relay in neurogenic inflammation (21). There is also an important link between emotional stress and the onset or exacerbation of acne (22) and the role of *C. acnes* in this context is widely discussed. *C. acnes* shows limited tolerance to oxygen and an affinity for lipidic environments. It therefore essentially colonizes skin niches, such as hair follicles and sebaceous glands, where it can form biofilms and develop at the immediate vicinity of capillary vessels and nerve terminals (23). Skin neuropeptides and hormones can diffuse throughout this microenvironment (21) and this motivated the study of the effect of local skin factors, such as natriuretic peptides, on the growth and biofilm formation of *C. acnes* (20). However, the effect of epinephrine and norepinephrine on *C. acnes* has not been formally investigated, although it has been mentioned as unpublished data in a recent review (6). In addition, *C. acnes* is a highly heterogeneous species and a precise study would require differentiating the responses of acneic and non-acneic strains, as clearly defined by Fitz-Gibbon et al. (24).

Here, we compared the effect of epinephrine and norepinephrine on the growth, biofilm formation, surface properties, membrane integrity, and size of acneic and non-acneic strains of *C. acnes*. The effect of catecholamines on the virulence of *C. acnes* was investigated on cultured sebocytes to mimic its natural environment. In addition, we evaluated the effect of thermal spring water from Uriage-les-Bains (UTW) on the response of *C. acnes* to catecholamines, as we previously observed that it was able to interfere with *C. acnes* biofilm formation (25).

## Materials and Methods

### *Cutibacterium* Strains and Culture Conditions

The acneic strain ribotype 4 (RT4) HL045PA1/HM-516 and non-acneic strain ribotype 6 (RT6) HL110PA3/HM-554 of *Cutibacterium acnes* [former *Propionibacterium acnes* and initially isolated by Fitz-Gibbon (24)] were obtained from BEI Resources American Type Culture Collection (Virginia, United States). These strains are of phylotypes IA1 and II, respectively (26). Bacteria stored at −80°C were initially plated on agar brain-heart infusion (BHI, BD), as recommended by BEI resources. As these strains are anaerobic, the plates were incubated under anoxic conditions at 37°C using a BD GasPack™ System or Whitley A85 Workstation.

Colonies were transferred into sterile conical 15-mL tubes (Falcon) filled to maximal capacity with reinforced clostridial medium (RCM) and grown at 37°C. As previously demonstrated, RCM was better adapted for the culture of these strains (27). Bacteria were collected after 72 h (stationary phase) and sub-cultured at 37°C under anoxic conditions in RCM supplemented, or not, with 10^−6^M epinephrine or norepinephrine (Sigma). Growth [medium optical density (OD)] was monitored using a Xenius XMA microplate reader (SAFAS). Inoculum was added in 96-well flat-bottom polystyrene plates (NUNC) at an initial OD_580_ = 0.08. Peripheral wells were filled with a CO_2_-producing solution. Plates were prepared under anoxic conditions and sealed with Parafilm before incubation under constant agitation. Optical density of the cultures was determined automatically every 15 min. Growth curves were determined over a minimum of three independent experiments.

### Measurement of Biofilm Formation Activity by Crystal Violet Staining

Biofilms were grown in 96-well flat-bottom polystyrene microtiter plates (NUNC) in the absence of agitation. Experiments were carried out according to the modified classical procedure (28). Bacteria were harvested (7,000 × g, 10 min) and rinsed with sterile physiological water [NaCl 0.9% (PS)]. An aliquot of 100 μL bacterial culture, adjusted to an OD_580_ = 1, was transferred to the wells of the microtiter plates and the plates incubated for 2 h to allow primary adhesion. Then, wells were washed with PS and fresh RCM, and the tested molecules added, or not. Plates were incubated for 72 h under static, anoxic conditions. At the end of the incubation, wells were washed four times with PS to remove planktonic bacteria. The biofilms were fixed with methanol for 15 min. After fixation, the methanol was removed, and the plates were dried and stained with 0.1% crystal violet (CV) for 10 min. After rinsing with PS, the dye was recovered by the addition of 100 μL acetone:ethanol (20:80, v/v), and the OD_595_ of the solution measured using a TECAN spectrophotometer. Ten wells were used for every experimental condition and each experiment was repeated a minimum of three times.

### Confocal Laser Scanning Microscopy

Biofilms were grown in 24-well-plates with a flat glass bottom (Sensoplate, Greiner bio-one, Germany). Bacteria were harvested (7,000 × g, 10 min) and rinsed with PS to remove all traces of medium. An aliquot of 300 μL bacterial culture, adjusted to an OD_580_ = 1, was transferred to the wells of the plates and the plates incubated for 2 h to allow primary adhesion. Then, the wells were washed with PS, media was added, and the plates were incubated for 72 h under static and anoxic conditions. At the end of the incubation, the wells were washed twice with PS to remove planktonic bacteria and the biofilms were stained with SYTO 9 Green Fluorescent Nucleic Acid Stain (Thermofisher^®^). Stained samples were fixed with ProLong Diamond Antifade Mountant (Molecular Probes^®^) and examined under an LSM 710 inverted confocal laser scanning microscope (Zeiss^®^, Germany) using the Zen^®^ 2009 software package. The Image J software package was used for mathematical analysis of the images and calculation of the parameters. Maximal and average biofilm thickness (μm) and their biomass volume (μm^3^/μm^2^) were determined. Each study was repeated a minimum of three times.

### Characterization of the Surface Polarity of *Cutibacterium acnes*

RT4 acneic and RT6 non-acneic strains of *C. acnes* grown in RCM supplemented, or not, with epinephrine or norepinephrine were collected in early stationary phase. Bacteria were harvested (7,000 x *g*, 10 min) and washed twice in PS. The surface polarity and Lewis acid-base balance of the bacteria were assessed using the Microbial Adhesion To Solvents (MATS) technique (29) and two solvent couples: chloroform/hexadecane and ethyl acetate/n-decane. For each bacterial strain and growth condition, 2.6 mL bacterial suspension at OD_400_ 0.8 was mixed for 60 s with 0.4 mL of each solvent. The tubes were vigorously shaken and after 15 min, the OD of the aqueous phase was measured at 400 nm. Controls were performed with the bacterial suspensions only. The percentage of cells in each solvent was calculated using the equation: % affinity = (1–A/A_0_) × 100, where A is the OD_400_ in the aqueous phase after incubation and Ao the OD_400_ of the control in the aqueous phase. Experiments were carried out in at least five replicates.

### Flow Cytometry Studies

RT4 acneic and RT6 non-acneic strains of *C. acnes* grown in RCM supplemented, or not, with epinephrine or norepinephrine were collected in early stationary phase. Bacteria were analyzed using a Beckman Cytoflex flow cytometer. Data were acquired for forward scatter (FSC) and side scatter (SSC) from 100,000 events per sample to study the size and granularity of the bacteria.

Cytoplasmic membrane integrity was assessed using a Molecular Probe™ LIVE/DEAD™ *Bac*Light™ Bacterial Viability Kit (Invitrogen) according to the manufacturer's instructions. Briefly, bacteria were harvested (7,000 × g, 10 min) and washed twice in PS. Propidium iodide (PI) labeled bacteria with damaged membranes red, whereas intact cells appeared green following labeling with SYTO 9. Flow cytometry controls (instrument set up, compensation, and gating) consisted of bacterial suspensions containing untreated and/or unlabeled cells and/or dead cells (treated with 50% ethanol for 1 h). Experiments were carried out in at least four replicates.

### Cytotoxicity Studies

The cytotoxic potential of acneic and non-acneic strains of *C. acnes* following, or not, exposure to epinephrine or norepinephrine was determined using a SZ95 human sebaceous gland cell line (30). Cell death was measured by lactate dehydrogenase (LDH) release into the medium by SZ95 sebocytes after exposure to control or treated bacteria. SZ95 cells were grown at 37°C in a 5% CO_2_ atmosphere in Sebomed basal Medium (Biochrom, F8205) supplemented with 10% inactivated fetal calf serum, 5 ng/mL h-EGF, 1 mM calcium chloride, and 50 μg/mL gentamycin. Cells were used between passages 19 and 35. They were seeded in 24-well plates and grown for 48 h before use. A minimum of 8 h before interaction with bacteria, cells were starved of antibiotics and fresh serum-free medium was added. Bacteria, grown in the presence or absence of catecholamines as previously described, were harvested (7,000 × g, 10 min), washed in PS, and used to infect SZ95 cells at a bacteria-to-cell ratio (MOI) of 50:1. The amount of LDH released by SZ95 cells was determined after 18 h of incubation using the Pierce LDH Cytotoxicity Assay Kit (Thermo Scientific, USA). Control studies using bacteria alone showed that none of the strains used in the present study produced metabolites that interfere with the assays. Experiments were carried out on at least three replicates.

### Interleukin 8 Secretion Study

The inflammatory response of SZ95 cells to acneic and non-acneic strains of *C. acnes* grown in RCM supplemented, or not, with epinephrine or norepinephrine was evaluated by assaying interleukin 8 (IL8) secretion into the culture medium. SZ95 cells were exposed to bacteria as already described. The amount of IL8 released by SZ95 cells was determined after 18 h of incubation using a human IL-8 ELISA Kit (KHC0081) (Invitrogen, ThermoFisher Scientific) according to the manufacturer's protocol. Experiments were carried out on at least three replicates.

### Oil Red O Staining and Lipid Detection

Acne is generally associated to hyperseborrhea. We thus investigated the impact of acneic and non-acneic *C. acnes* exposed, or not, to catecholamines on sebocyte lipid production. The amount of general lipids synthesized by SZ95 cells was evaluated by Oil Red O staining. Briefly, SZ95 cells were exposed to bacteria as already described, except that 96-well-plates were used. After 18 h of incubation, cells were washed twice with phosphate buffer (0.1M, pH 7.4) and fixed with 4% paraformaldehyde (Acros Organics) for 10 min. Fixed cells were washed with 60% isopropanol (Merck) and stained with a 0.5% Oil Red O staining solution made in isopropanol (Sigma-Aldrich):distilled water (6:4, v/v). For quantitative detection of intracellular lipids, water was removed from the Oil Red O-stained cells and Oil Red O was then eluted by incubating the cells with 100% isopropanol for 5 min. After pipetting several times, to ensure that all Oil Red O had been eluted, the concentration of Oil Red O in the supernatant was evaluated by measuring the optical density at 500 nm. Experiments were carried out on at least four replicates.

### *In silico* Studies

The genome of (RT4) HL045PA1/HM-516 and (RT6) HL110PA3/HM-554 *C. acnes* strains has been sequenced (24) and the bacterial catecholamine receptor, Qsec, has been previously identified in *E. coli* (4, 31). We thus used an *in-silico* approach to investigate the presence of a catecholamine receptor in *C. acnes*. All calculations were performed using a DELL PowerEdge T420 computer equipped with four hard disks (4 To each, for a total of 12 To under RAID5). The FASTA amino-acid sequence of the *C. acnes* genomes was aligned by BLASTp on ExPASy (https://web.expasy.org/blast) with the sequence of the *E. coli* two-component system sensor histidine kinase QseC (GenBank: RZN88374.1). Protein 3D models were generated using RaptorX Structure (32) and visualized using Python Molecular Viewer V1.5.6.Prediction by alignment on the crystalized *E. coli* QseC structure. The potential binding of ligands to the identified sensor protein was studied using AutoDock 4.2 (33). Binding values were generated using the Lamarkian Genetic Algorithm of AutoDock 4.2.

### Statistical Analysis

The data were analyzed using the Mann-Whitney non-parametric test as they were not normally distributed (Gaussian). Microsoft Excel 2007 (R software) was used to generate graphs and determine confidence coefficients. Statistical significance was based on alpha = 0.05 and is indicated on the figures by a star. Higher confidence values (*p* < 0.01 and *p* < 0.001) are indicated by two or three stars, respectively.

## Results

### Epinephrine and Norepinephrine Stimulate Biofilm Production by *Cutibacterium acnes*

**Figure 1:**
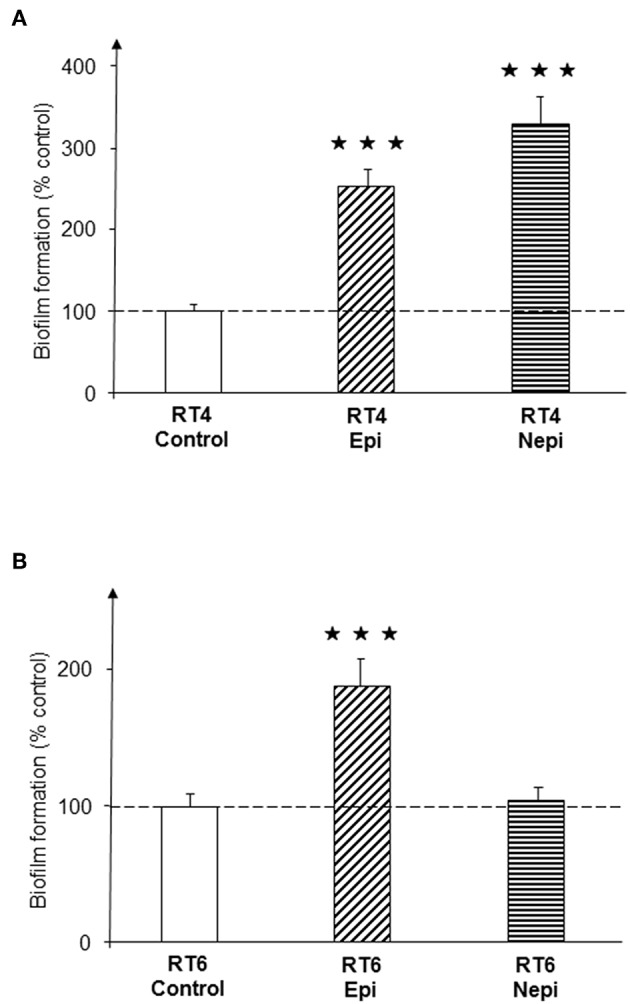
Effect of epinephrine and norepinephrine on biofilm formation by acneic and non-acneic strains of *Cutibacterium acnes* in polystyrene microtiter plates. The effect of epinephrine (Epi) or norepinephrine (Nepi) (10^−6^ M) on biofilm formation by the RT4 acneic **(A)** and RT6 non-acneic strains **(B)** of *C. acnes* was measured by crystal violet staining. Results are shown as the mean ± SEM of a minimum of three independent experiments (^⋆⋆⋆^*p* < 0.001).

A preliminary study performed over 72 h of culture in RCM showed that epinephrine and norepinephrine (10^−6^ M) had no effect on the grown of the RT4 acneic and RT6 non-acneic strains of *C. acnes*. Bacteria were then grown in polystyrene microtiter plates to investigate the effect of the catecholamines on biofilm formation using the crystal violet technique. Epinephrine and norepinephrine induced a significant increase in biofilm formation by the RT4 acneic strain after 72 h of culture under static conditions (153 ± 20 and 227 ± 36%, respectively, *p* < 0.001) (Figure 1A). Epinephrine also increased the formation of biofilm by the RT6 non-acneic strain (88 ± 19%, *p* < 0.001), but norepinephrine had no effect on this strain (*p* = 0.76) (Figure 1B).

**Figure 2:**
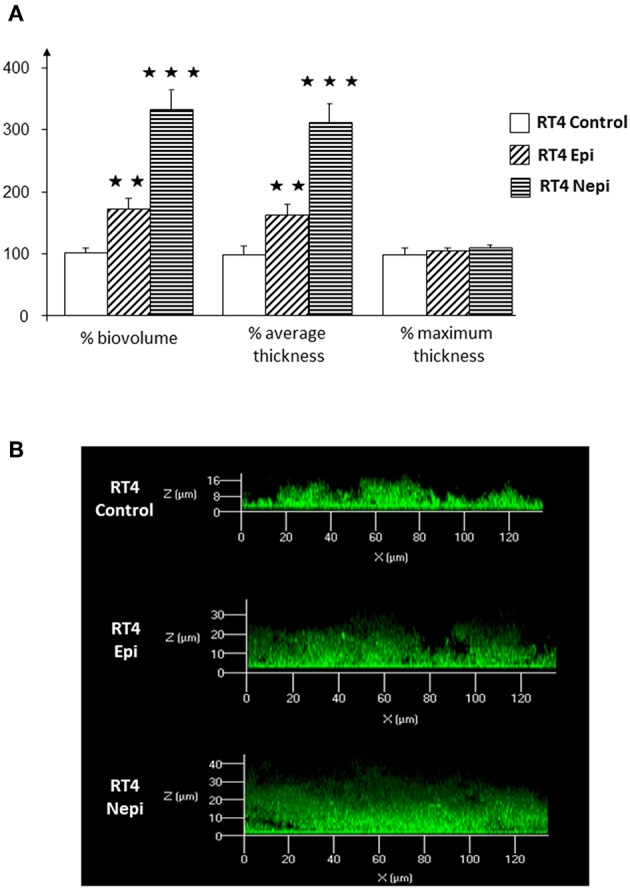
Effect of epinephrine and norepinephrine on biofilm formation by the RT4 acneic strain of *Cutibacterium acnes* on a glass support measured by confocal laser scanning microscopy. **(A)** Biovolume, average thickness, and maximum thickness of the biofilms after exposure to epinephrine (Epi) or norepinephrine (Nepi) calculated using Zen^®^ 2009 software. **(B)** Representative views of X/Z sections of the biofilms formed in the presence or absence of epinephrine (Epi) or norepinephrine (Nepi). (^⋆⋆^*p* < 0.01, ^⋆⋆⋆^*p* < 0.001).

**Figure 3:**
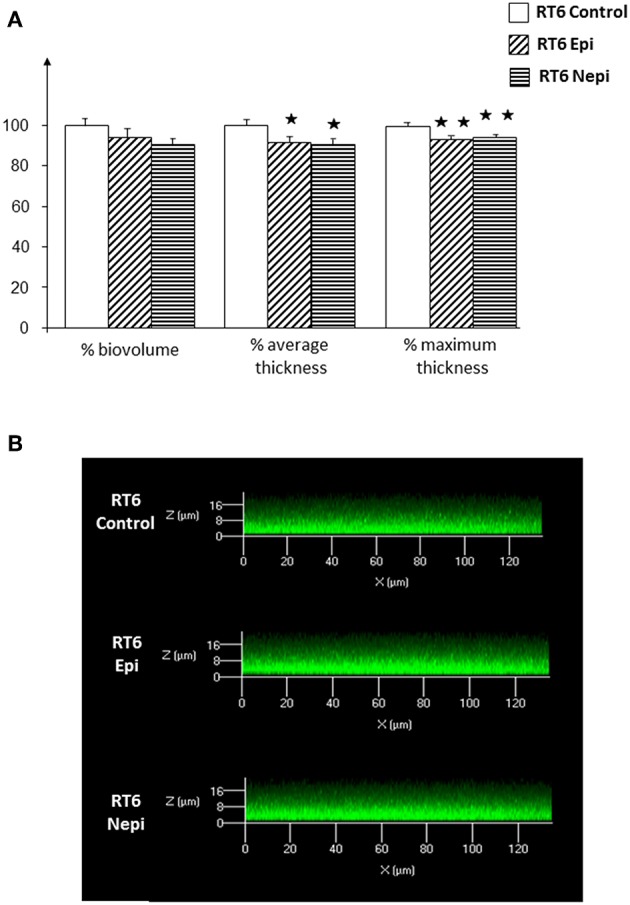
Effect of epinephrine and norepinephrine on biofilm formation by the RT6 non-acneic strain of *Cutibacterium acnes* on a glass support measured by confocal laser scanning microscopy. **(A)** Biovolume, average thickness, and maximum thickness of the biofilms after exposure to epinephrine (Epi) or norepinephrine (Nepi) calculated using Zen^®^ 2009 software. **(B)** Representative views of X/Z sections of the biofilms formed in the presence or absence of epinephrine (Epi) or norepinephrine (Nepi). (^⋆^*p* < 0.05, ^⋆⋆^*p* < 0.01).

We then investigated the structure of the biofilm by confocal microscopy. Biofilms formed by the RT4 acneic strain were very heterogeneous, of highly varying thickness. Confocal microscopy confirmed the effects of the catecholamines on biofilm formation by the RT4 acneic strain. The average thickness of the biofilm increased significantly after exposure of the bacteria to epinephrine (60 ± 18%, *p* < 0.01) and norepinephrine (211 ± 29%, *p* < 0.001) (Figure 2). The mean biovolume of the biofilm calculated by Image J software increased by the same proportions (70 ± 20% in the presence of epinephrine, *p* < 0.01, and 232 ± 30% in the presence of norepinephrine, *p* < 0.001). Only the maximum thickness did not change, probably because of the very irregular structure of the biofilm formed by this bacterium. Biofilms formed by the RT6 non-acneic strain were markedly different, as they were remarkably homogeneous (Figure 3). Norepinephrine had no effect on the mean biovolume of the biofilms formed by this strain, by crystal violet staining. However, there was a small, but significant, decrease of the mean and maximal thickness, although the significance of this result is probably due to the highly homogeneous nature of the biofilm and does not reflect a true physiological response. There was a marked difference between the results obtained by confocal microscopy and those by crystal violet staining for the effect of epinephrine on biofilm formation by the RT6 strain, as there was a total absence of a stimulatory effect. Indeed, epinephrine showed essentially the same effect as norepinephrine, with a parallel minor decrease of the mean and maximal biofilm thickness. This difference may be related to the surface on which the biofilms were grown between the two techniques, i.e., polystyrene, a hydrophobic surface, and glass, which is hydrophilic.

### Norepinephrine Modulates the Surface Polarity of *Cutibacterium acnes*

**Figure 4:**
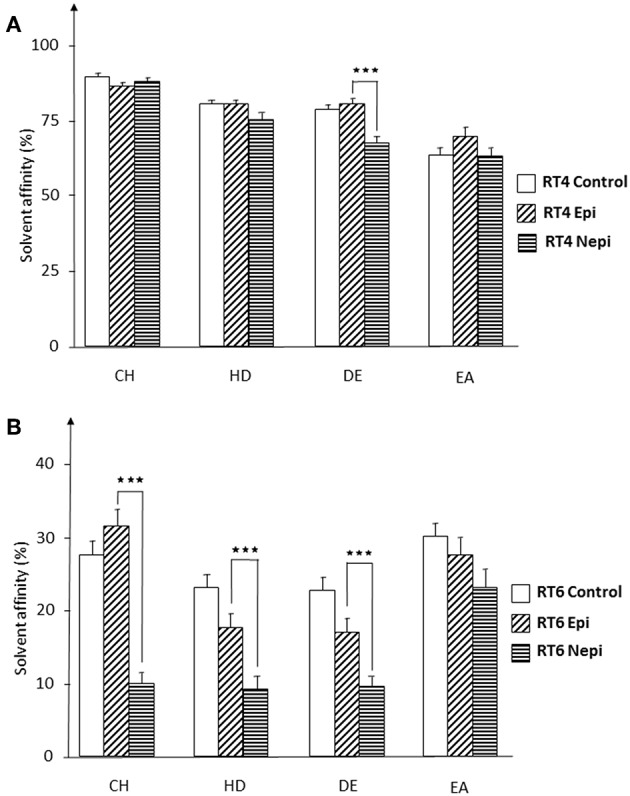
Affinity of RT4 acneic **(A)** and RT6 non-acneic **(B)** strains of *Cutibacterium acnes*, grown in the presence or absence of epinephrine (Epi) or norepinephrine (Nepi) (10^−6^ M), for solvents. The partitioning of RT4 and RT6 strains of *C. acnes* between water and solvents of various polarities was studied by the MATS technique using chloroform (CH), hexadecane (HD), decane (DE), and ethyl acetate (EA). Results are representative of five independent experiments. (^⋆⋆⋆^*p* < 0.001).

The differences in the response of the RT4 and RT6 *C. acnes* biofilms to catecholamines appear to depend on the surface on which they are grown. Thus, we performed a MATs test on the bacteria to determine their mean surface polarity. The RT4 acneic strain showed a very high affinity for all solvents, suggesting that this strain has a highly hydrophobic surface (Figure 4A). This is coherent with its relatively lower affinity for ethyl acetate, the most polar solvent of the series. Although, epinephrine and norepinephrine markedly affected biofilm formation by this strain, the catecholamines had limited effect on its surface polarity. The affinity of epinephrine treated RT4 *C. acnes* to the four solvents was not significantly different from that of control bacteria. Even norepinephrine, which had a stronger effect on biofilm formation, only minimally effected the surface polarity of this strain. Only the affinity for decane was reduced, suggesting a decrease of the Lewis basic character of its surface. The RT6 non-acneic strain showed completely different surface properties (Figure 4B). This strain was more polar than RT4 and showed limited affinity for all solvents. Epinephrine stimulated biofilm formation by the RT6 non-acneic strain, as shown by crystal violet staining, but there was no significant alteration of its affinity for solvents and thus surface polarity. In contrast, norepinephrine, which showed a very limited effect on biofilm formation by this strain, markedly reduced the affinity of the bacterium to chloroform, hexadecane, and decane, suggesting reinforcement of the polar character of its surface. Only the affinity for ethyl acetate showed a non-significant reduction. These results show that there is no association between the effect of catecholamines on biofilm formation and the mean surface polarity of the bacterial population or that any association is very limited.

### Catecholamines Have no Influence on the Membrane Integrity, Viability, Surface Granularity, or Size of *C. acnes*

**Figure 5:**
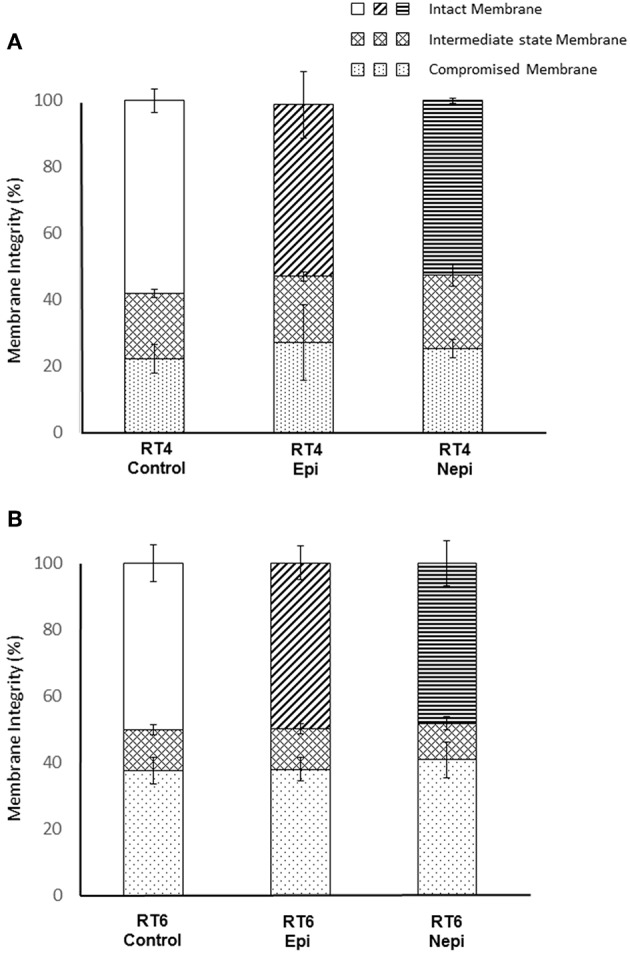
Evaluation of membrane integrity of RT4 acneic **(A)** and RT6 non-acneic **(B)** strains of *Cutibacterium acnes* grown in the presence or absence of epinephrine (Epi) or norepinephrine (Nepi) (10^−6^ M). Membrane integrity was classified as “intact,” “intermediate state,” or “membrane” following labeling using the LIVE/DEAD™ *Bac*Light™ Bacterial Viability Kit and flow cytometry analysis. Results are shown as the mean ± SEM of data acquired from 100,000 events.

Our results suggest that factors other than the surface polarity of the bacteria are involved in the effects of catecholamines on biofilm formation. Thus, we examined the RT4 acneic and RT6 non-acneic strains of *C. acnes* by flow cytometry using the LIVE/DEAD™ *Bac*Light™ bacterial viability fluorescent test. In addition to viability, this test provides data on the integrity of the bacterial envelope, as it is based on the penetration of all calls by a green fluorescent dye and that of bacteria showing alterations in membrane integrity by a red fluorescent dye, here propidium iodide. A mean of 40% of RT4 acneic *C. acnes* showed moderately compromised membranes (Figure 5A). This would appear to be important, especially as these bacteria were collected in early stationary phase. However, this may also reflect the difficulty of adapting this strain to artificial growth medium. Exposure to catecholamines had no significant effect on the membrane integrity of these bacteria, although we observed a marginal decrease, presumably associated with stress. The results for the RT6 non-acneic strain were similar (Figure 5B). The percentage of bacteria with a completely functional membrane was even lower (approximately 50%) and exposure to epinephrine or norepinephrine had no effect on membrane integrity or the viability of *C. acnes*. Plots of the flow cytometry data of the surface granularity vs. bacterial size showed that neither of these parameters of the acneic and non-acneic strains of *C. acnes* were altered by exposure to catecholamines (Supplementary Data 1).

### Catecholamines Do Not Increase the Intrinsic Cytotoxicity or Inflammatory Potential of *C. acnes* on Sebocytes

**Figure 6:**
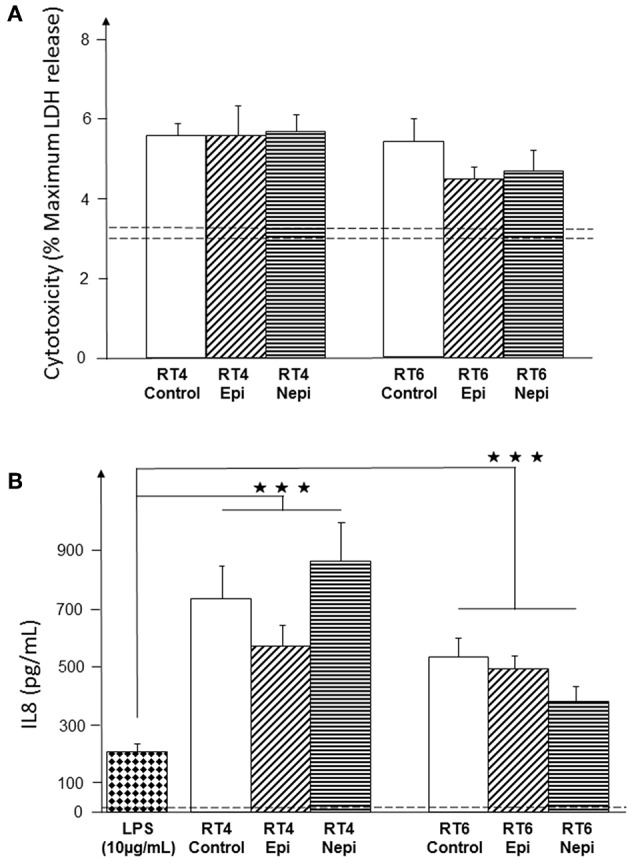
Effect of epinephrine and norepinephrine on the cytotoxicity and inflammatory potential of RT4 acneic and RT6 non-acneic strains of *C. acnes*. Cytotoxicity **(A)** was measured by LDH released by SZ95 sebocytes after exposure to bacteria. Cytotoxicity is expressed as the percentage of maximal LDH release obtained by total lysis of cultured cells using Triton X100. Dotted lines indicate the mean spontaneous cell death (percentage) in control cultures. The inflammatory response of SZ95 sebocytes after exposure to bacteria **(B)** was measured by interleukin 8 (IL8) released into the culture medium. IL8 secretion was very low under control conditions (dotted line). SZ95 sebocytes were exposed to lipopolysaccharide (LPS) (10 μg/mL) as a positive control for the inflammatory response. Results are the shown as the mean ± SEM of a minimum of three independent experiments (^⋆⋆⋆^*p* < 0.001).

The influence of host factors on the virulence of cutaneous bacteria is generally investigated using keratinocytes. However, the natural environment of lipophilic and anaerobic bacteria, such as *C. acnes*, is the sebaceous gland. Thus, we investigated the effect of catecholamines on *C. acnes* cytotoxicity and inflammatory potential using cultured sebocytes. Both acneic and non-acneic RT4 and RT6 strains showed equivalent cytotoxic activity, as measured by LDH release (Figure 6A), which was low and only marginally higher than that of basal cell death in the cultures. Neither epinephrine nor norepinephrine modified the cytotoxicity of either strain. We also measured IL8 production by sebocytes as a marker of inflammation, as acne is more highly associated with inflammation than cell death. The basal production of IL8 by the SZ95 sebocyte cell line was low (14 pg/mL). However, these cells were able to react to the inflammatory substance LPS (10 μg/mL) by markedly increasing IL8 secretion (Figure 6B). Both *C. acnes* strains had a stimulatory effect on IL8 production by sebocytes and, as expected, the acneic strain showed higher inflammatory potential than the non-acneic strain. However, the effects of epinephrine and norepinephrine on IL8 production were not statistically significant (all *p* values > 0.05).

### Catecholamine Treatment of the RT4 Acneic Strain of *C. acnes* Stimulates Lipids Production by Sebocytes

**Figure 7:**
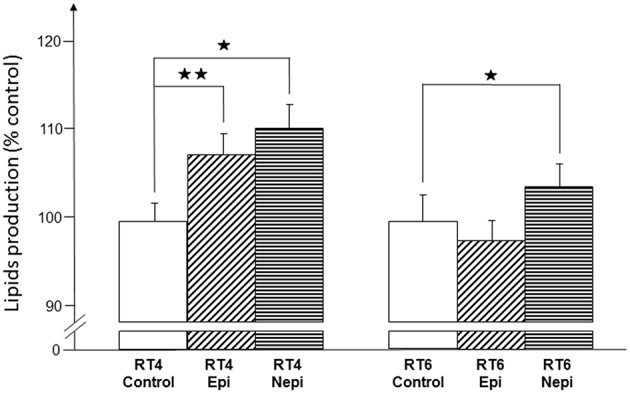
Effect of epinephrine and norepinephrine on the potential of RT4 acneic and RT6 non-acneic strains of *C. acnes* to induce lipid production by sebocytes. Lipid production by SZ95 sebocytes was measured by the Oil Red O technique after an 18-h incubation with control or epinephrine (Epi) or norepinephrine (Nepi) treated bacteria. Results are shown as the mean ± SEM of a minimum of three independent experiments (^⋆^*p* < 0.05, ^⋆⋆^*p* < 0.01).

We investigated the potential effect of acneic and non-acneic *C. acnes* exposed, or not to catecholamines, on lipid production by measuring the total amount of lipids produced by SZ95 sebocytes using the Oil Red O technique. Under our culture conditions, neither the RT4 acneic nor RT6 non-acneic strains of *C. acnes* had an effect on the lipid production of SZ95 sebocytes (Figure 7). However, epinephrine- and norepinephrine-treated RT4 acneic *C. acnes* induced a limited but significant increase of lipid production (7 ± 2% and 10 ± 3%, respectively). Interestingly, we observed this effect after a short period of exposure (18 h) relative to that in real life. Norepinephrine treated RT6 non-acneic *C. acnes* also induced a minor but significant increase in lipid production, whereas epinephrine had no effect.

### Effect of Uriage Thermal Spring Water on *C. acnes* Biofilm Formation and Its Response to Epinephrine and Norepinephrine

We investigated the effect of epinephrine and norepinephrine on RT4 acneic and RT6 non-acneic strains of *C. acnes* in medium (RCM) supplemented with 30% Uriage thermal water (UTW), a natural mineral water recognized by the French Academy of Medicine in 1877 for its positive effects on inflammatory diseases and used to treat cutaneous inflammatory diseases, such as acne. Controls consisted of RCM diluted with 30% physiological water (PS, 0.9% NaCl). These studies were followed by crystal violet staining and confocal microscopy.

**Figure 8:**
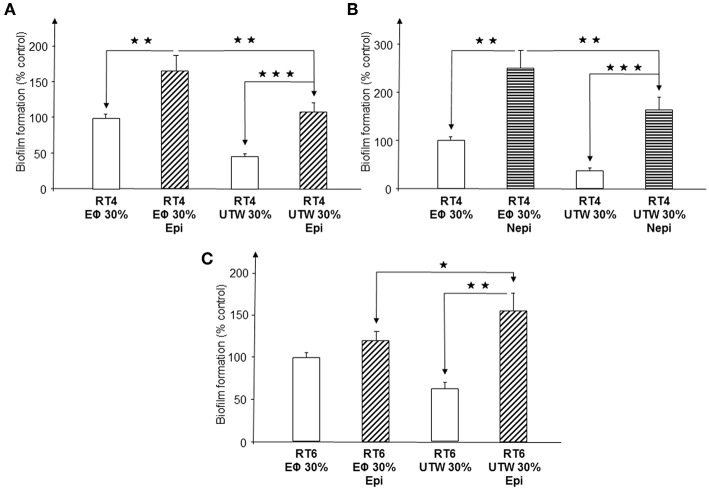
Influence of Uriage thermal water on biofilm formation by acneic and non-acneic strains of *Cutibacterium acnes* in the presence of absence of epinephrine or norepinephrine in polystyrene microtitration plates. The influence of Uriage thermal water (30% UTW in growth medium) on biofilm formation by RT4 acneic **(A,B)** and RT6 non-acneic **(C)** strains of *C. acnes* in the presence or absence of epinephrine (Epi) or norepinephrine (Nepi) (10^−6^ M) was measured by crystal violet staining. Physiological water (Eϕ; 0.9% NaCl, 30% in growth medium) was used as a control. Results are shown as the mean ± SEM of a minimum of three independent experiments. (^⋆^*p* < 0.015, ^⋆⋆^*p* < 0.01, ^⋆⋆⋆^*p* < 0.001).

Exposure of the RT4 acneic strain grown in medium containing 30% PS to epinephrine provoked a marked stimulation of biofilm formation, measured by crystal violet staining (66 ± 22%, *p* < 0.01) (Figure 8A). Growth of the bacteria in medium supplemented with 30% UTW resulted in lower basal production of biofilm and exposure to epinephrine resulted in biofilm formation that remained significantly lower than that of bacteria grown in medium containing 30% PS and within the control values (*p* < 0.01). The response of RT4 *C. acnes* to norepinephrine was highly similar, except that the stimulation of biofilm formation induced by this catecholamine in the presence of 30% PS medium was higher (149 ± 37%, *p* < 0.01) and 30% UTW could not completely inhibit the effect of norepinephrine, with a 64 ± 27% stimulation of biofilm formation under this condition (Figure 8B). We tested epinephrine only on the RT6 non-acneic strain in the presence of UTW, since norepinephrine was inactive on this strain. Epinephrine did not significantly stimulate biofilm formation by this strain in medium supplemented with 30% PS (Figure 8C). UTW decreased RT6 biofilm formation but exposure of the bacterium to epinephrine in the presence of 30% UTW induced a limited but significant increase of the biofilm (55 ± 21%, *p* < 0.05).

**Figure 9:**
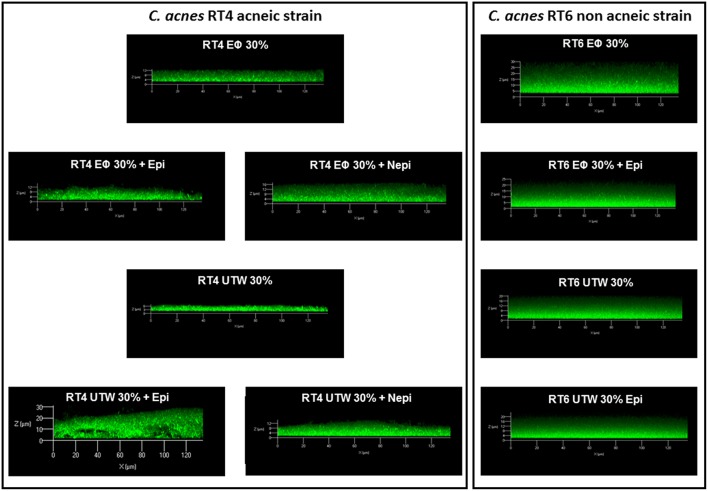
Structure of acneic and non-acneic *Cutibacterium acnes* biofilms formed in the presence of Uriage thermal water or physiological water in the presence or absence of epinephrine or norepinephrine. The structure of biofilms formed by RT4 acneic and RT6 non-acneic strains of *C. acnes* in the presence of Uriage thermal water (UTW) or physiological water (Eϕ) (30% in growth medium) and the presence or absence of epinephrine (Epi) or norepinephrine (Nepi) (10^−6^ M) was visualized by confocal laser scanning microscopy. All figures are represented at the same scale and show a X/Z scan of the biofilms (mean thickness).

Confocal microscopy gave partially different results. As observed by crystal violet staining, norepinephrine stimulated biofilm formation by the RT4 acneic strain in the presence of PS but not in the presence of UTW (Figure 9). UTW also showed an intrinsic inhibitory effect on RT4 *C. acnes* biofilm formation relative to that of PS. In contrast, we did not observe the stimulatory effect of epinephrine in the presence of PS but it appeared to be enhanced in the presence of UTW. The results for the RT6 strain grown in medium supplemented with PS or UTW, in the absence or presence of epinephrine, were almost identical to those previously obtained on the same surface, with no essential variation of biofilm thickness or structure.

### Identification of a Potential Catecholamine-Binding Protein in the *C. acnes* Genome

**Figure 10:**
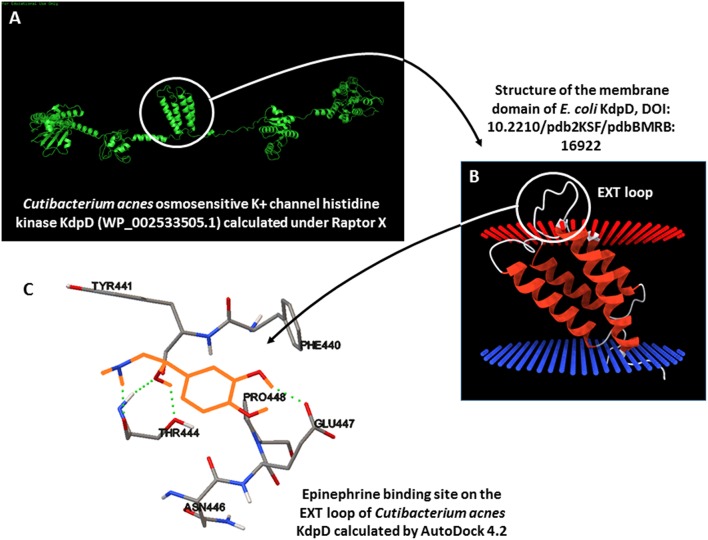
3D modeling of the potential catecholamine-binding site on *Cutibacterium acnes* KdpD. **(A)** Calculation of the 3D structure of *C. acnes* KdpD using RaptorX. **(B)** Organization of the central membrane domain of KdpD, showing the EXT external loop. **(C)** Calculated epinephrine-binding site on *C. acnes* KdpD determined using AutoDock 4.2 (epinephrine is shown in orange).

BLASTp studies did not reveal the presence of an ortholog of QseC, the *E. coli* catecholamine receptor, in the genome of the *C. acnes* RT4 and RT6 strains. However, an osmosensitive K^+^ channel histidine kinase, KdpD (NCBI reference sequence WP_002533505.1), showed partial homology with QseC. Moreover, this KdpD protein has a particular structure, as it is almost double the size of *E. coli* QseC and its organization suggests that it resulted from a gene duplication and inversion. On the basis of the UNIPROT Q6ABI9 sequence of *C. acnes* (strain DSM 16379 / KPA171202), we generated a 3D model using RaptorX Structure Prediction by alignment on the crystalized *E. coli* QseC structure. The association of epinephrine, norepinephrine, and phentolamine [a reversible non-selective α-adrenergic antagonist, (34)] to kdpD using AutoDock 4.2 revealed potential binding of epinephrine in the EXT outer-loop region of KdpD between amino-acids 426 and 426 (Figure 10). The calculated binding values between epinephrine, norepinephrine, and phentolamine were −4.47, −4.97, and −5.6 Kcal/mol respectively, indicating a likely interaction between catecholamines and this EXT loop of KdpD. Indeed, the principal amino acids involved in the binding of catecholamines to KdpD determined by molecular docking (Phe 440, Tyr441, Thr444, Asn446, Glu 447, and Pro448 for epinephrine and Phe440, Tyr441, Thr444, Glu447, and Pro448 for norepinephrine) are almost the same as those previously determined for the binding of catecholamines in a eukaryotic model (RCSB-PDB 2QEO).

## Discussion

Here, weprovide the first detailed study and demonstration of the effect of catecholamines on *C. acnes*. Epinephrine and norepinephrine had no effect on the growth of *C. acnes*, as already observed for other neurohormones encountered in skin, such as natriuretic peptides (20), perhaps explaining why this interaction between host and bacteria has been largely ignored. In addition, the diversity of *C. acnes* requires the precise identification of strains representative of the acneic and non-acneic forms of this species, such as the RT4 and RT6 strains used herein.

Epinephrine is essentially synthesized by the adrenal medulla and released into the blood from where it reaches multiple targets, including the skin, and acts as one of the principal stress hormones (35). Conversely, norepinephrine is released principally by sympathetic nerve terminals and, as previously mentioned, is one of the major cutaneous neurotransmitters (13, 14). Thus, epinephrine and norepinephrine in the skin originate from different sources. This is particularly intriguing concerning the difference of sensitivity of the two strains of *C. acnes* to these molecules. Indeed, the RT4 acneic strain showed a strong reaction to both catecholamines in terms of biofilm formation, whereas the RT6 non-acneic strain appeared to be only sensitive to epinephrine. In addition, the stimulation of biofilm formation induced by epinephrine was lower for the RT6 strain than the RT4 strain (153 vs. 60%) and was only observed when the cells were grown on a hydrophobic surface, such as polystyrene. The RT4 acneic strain is adapted to a hydrophobic environment and is strictly anaerobic, whereas the RT6 non-acneic strain requires a more polar medium and is more aerotolerant (27). The RT4 acneic strain has been postulated to originate from the sebaceous compartment, whereas the RT6 non-acneic strain has been postulated to come from the skin surface and upper part of the hair follicle. Given these different ecological niches, the RT4 strain, found deep in the skin, should be in greater contact with catecholamines than the R6 strain, which is only exposed to molecules transported by sweat. The physiological concentrations of epinephrine and norepinephrine in sweat remains unknown (36) but, because of its location distant from nerve endings, the RT6 non-acneic strain should be naturally more exposed to epinephrine than norepinephrine. Thus, the high sensitivity of the RT4 strain to both catecholamines and the limited sensitivity of the RT6 strain to only one, i.e., epinephrine, should reflect an adaptation to the host microenvironment. In support of this hypothesis, the RT4 strain forms heterogeneous and fragile biofilms, whereas biofilms formed by the RT6 strain are dense and more resistant. Indeed, the structure of biofilms is influenced by the stability of the environment, as demonstrated in a flexible biofilm-forming species, such as *Vibrio cholera* (37). The RT4 strain, growing in the sebaceous gland under stable conditions, should produce a less resistant biofilm than the RT6 strain, which is exposed to the variations encountered on the skin surface. However, these differences probably do not reflect complete and definitive adaptation of the bacteria, as shown by MATs studies, since the RT6 strain is still sensitive to epinephrine and norepinephrine. The highly hydrophobic strain RT4 did not show major changes in surface polarity in response to catecholamines, suggesting that the effect of these molecules on biofilm formation are mediated through the expression of specific adhesins, as suggested by Holland et al. (38). Conversely, the RT6 strain showed a polar surface and the hydrophilic character of this bacterium increased after exposure to norepinephrine. This observation is consistent with an effect of catecholamines on specific adhesins, as this molecule had no effect on biofilm formation, despite the large changes of surface polarity induced by norepinephrine.

In Gram-positive bacteria, such as Staphylococci, variations of surface energy and roughness are associated with changes in viability (39). However, we observed no differences in membrane integrity or roughness of the two *C. acnes* strains, suggesting that the changes in biofilm formation and surface polarity induced by catecholamines are within the normal physiological range of adaptation of these bacteria. In our experimental model, *C. acnes* did not respond to catecholamines by an increase of virulence, in contrast to *E. coli* and *Citrobacter rotentium* (8). The cytotoxic activity of both bacteria was very low and showed no difference between the acneic and non-acneic strains. However, the cytotoxicity studies were performed using planktonic bacteria, as growing biofilms on cultured cells was not possible. As recently shown, the matrix of *C. acnes* contains highly diverse molecules, which can affect the viability of eukaryotic cells (40), and biofilm matrix is frequently considered itself to be a virulence factor (41). In addition, *C. acnes* virulence factor expression can be modulated by the microenvironment (27) and generally requires the contribution of host molecules, such as acid sphingomyelinase (42). However, *C. acnes* is not a true pathogen but more a cause of inflammation (38). We observed that SZ95 sebocytes are able to generate an inflammatory response after exposure to LPS, using interleukin 8 (IL8) as a marker of inflammation. Sebocytes also responded to both *C. acnes* strains by a marked increase of IL8 release, the acneic strain inducing greater IL8 production. However, the catecholamines had no effect. This does not exclude a potential role of catecholamines in *C. acnes* inflammatory activity, as other host molecules are likely necessary for full expression of this potential. These results are similar to those obtained using HaCat keratinocytes (27) and SZ95 sebocytes grow in a complex sebum-like medium which is particularly favorable to *C. acnes*. Thus, the low cytotoxicity and inflammatory potential of both strains of *C. acnes* suggest that this bacterium behaves more as a skin commensal than as an opportunistic pathogen, at least in the absence of stimulation by host factors. Indeed, epinephrine and norepinephrine showed a significant effect on the potential of the RT4 acneic strain to stimulate lipid production by sebocytes. In acne, hyperseborrhea plays a central role in pore occlusion, leading to the development of an anaerobic environment favorable for *C. acnes* development (43) and, as shown here, catecholamines appear to trigger this process by interacting with *C. acnes*. Interestingly, the RT6 non-acneic strain only stimulated lipid secretion in response to norepinephrine. As already discussed, this observation is coherent with the ecological niche of this strain on the skin surface, which limits its natural exposure to catecholamines.

Thermal water from many sources is known for its positive effect on chronic skin diseases. This is also true for thermal spring water from Uriage-les-Bains (UTW), recognized since 1877 by the French Academy of Medicine for its activity against inflammation, particularly acne. However, there have only been limited scientific studies on the effects of thermal water. We previously showed that UTW can inhibit the increase of virulence induced by substance P on *B. cereus, S. aureus*, and *S. epidermidis* (17) and more recently we reported that it can interfere with biofilm formation by *C. acnes* acneic strains (25). These studies, including this study, have all been carried out in the framework of a long-term industrial partnership validated by Rouen Normandy University and the doctoral school EdNBISE (Rouen and Caen universities), in which the industrial partner has no influence on the scientific results. Here, we confirm the inhibitory effect of UTW on biofilm formation by acneic strains of *C. acnes*. Crystal violet staining showed that UTW inhibited catecholamine-induced biofilm formation by the RT4 strain but that such inhibition was in the same range as that observed with UTW alone. This suggests that the inhibition resulted from the intrinsic effect of UTW on the basal level of biofilm production by the RT4 strain and not by a direct interaction with the action of the catecholamines. The surface on which the biofilms were formed also had a strong influence. Polystyrene surfaces, such as the microtiter plates used for the crystal violet studies, are hydrophobic, whereas glass surfaces used in confocal microscopy are polar and hydrophilic. This could explain the difference in the response to catecholamines of the RT4 strain between the two techniques. Indeed, as seen by the crystal violet technique, UTW inhibited biofilm formation by RT4 in the presence of norepinephrine, relative to physiological water, but increased the thickness of the biofilm formed in response to epinephrine. Moreover, we did not observe the effects of UTW on the response of the non-acneic strain RT6 to catecholamines on the glass used for confocal microscopy. These results confirm that UTW inhibits *C. acnes* biofilm formation but its effect on the response to catecholamines appears to be more limited and surface dependent. Clinical studies should be performed to validate the potential of UTW to inhibit catecholamine induced *C. acnes* biofilm formation.

Demonstration of the sensitivity of bacteria to host factors requires identification of the bacterial sensor or receptor. Such studies have led to the characterization of bacterial substance P (17), calcitonin gene related peptide (18), and even natriuretic peptide receptors (44). However, such studies are long and resource intensive. We decided to use a bioinformatic approach, as the genomes of the two *C. acnes* strains have been sequenced. Alignment of the deduced amino-acid sequences of the RT4 and RT6 *C. acnes* genomes with that of the *E. coli* catecholamine receptor QseC did not lead to the identification of an ortholog of this protein in *C. acnes*. However, another molecule, the osmosensitive K^+^ channel histidine kinase KdpD (NCBI reference sequence WP_002533505.1) showed partial homology with QseC. This KdpD protein has a peculiar structure, as it is almost double the size of *E. c*o*li* QseC and its organization suggests that it resulted from a gene duplication and inversion. A 3D model of KdpD, generated using RaptorX Structure Prediction by alignment on the crystalized *E. coli* QseC structure, showed the presence of a central core formed by four α-helix sequences, which in *E. coli* (KdpD is also present in the *E. coli* genome) are considered to be transmembrane domains and present on an EXT loop on the outer cytoplasmic side. Calculation of the binding values of catecholamines with this EXT loop revealed a likely interaction. Moreover, principal amino acids identified in the binding site are almost the same as those determined for the binding of catecholamines in a eukaryotic receptor model (RCSB-PDB 2QEO). These results need to be confirmed by biochemical studies but provide a first indication that *C. acnes* expresses a membrane sensor for catecholamines related to, but different from, that identified in *E. coli*.

Overall, our data show that biofilm formation by *C. acnes* and its effect on sebum production can be regulated by catecholamines. This finding merits clinical confirmation and suggests a new role for *C acnes* as a relay between stress and acne. Our results also explain, at least in part, how certain natural treatments, such as UTW, may act on acne.

## Data Availability

All datasets generated for this study are included in the manuscript and/or the Supplementary Files.

## Author Contributions

VB, PT, and CC performed the experiments, analyzed the data, and wrote the draft of the manuscript. P-JR performed the molecular-modeling studies. YK-G, LL, and CD-P supervised the work. CZ provided the sebocyte cell line. MF organized the funding, supervised the work, and assisted in writing and reviewing of the manuscript. All authors read and approved the final manuscript.

### Conflict of Interest Statement

LL is employed by the Dermatologic Laboratories Uriage. There are no patents, products in development of marketed products to declare. The remaining authors declare that the research was conducted in the absence of any commercial or financial relationships that could be construed as a potential conflict of interest.

## Supplementary Material

Supplementary Data 1 Flow cytometry graphs showing surface granularity vs. bacterial size for RT4 **(A,B)** and RT6 **(C,D)** C. acnes strains exposed to epinephrine (Epi) or norepinephrine (Nepi).
