# Supplementary figures and images for: Acne and Stress: Impact of Catecholamines on Cutibacterium acnes
Source: Front Med (Lausanne). 2019 Jul 10;6:155. doi: 10.3389/fmed.2019.00155 (PMC6635461; doi:10.3389/fmed.2019.00155)

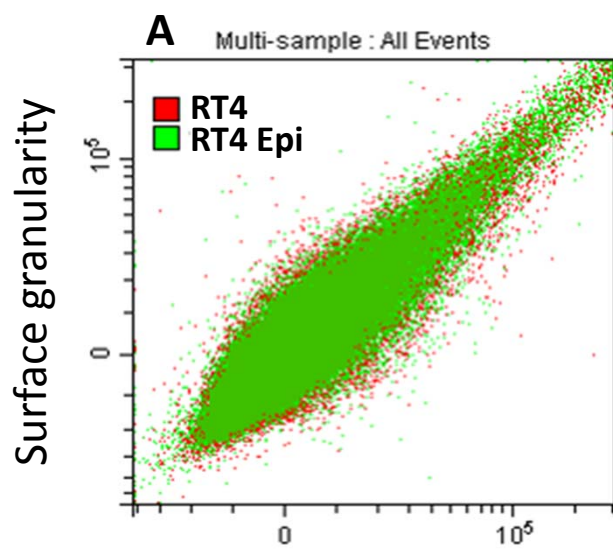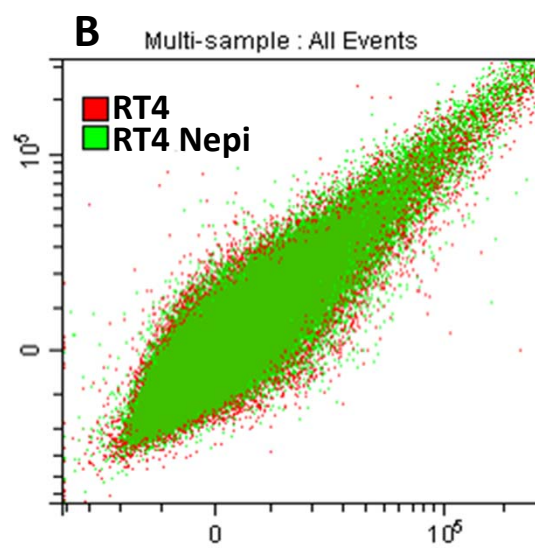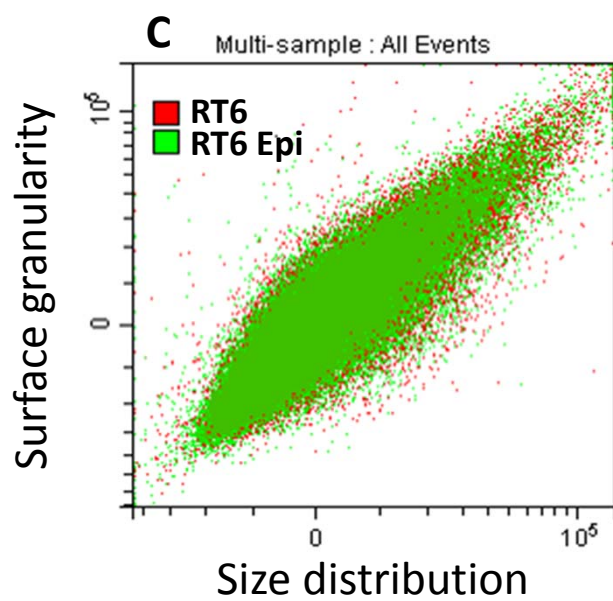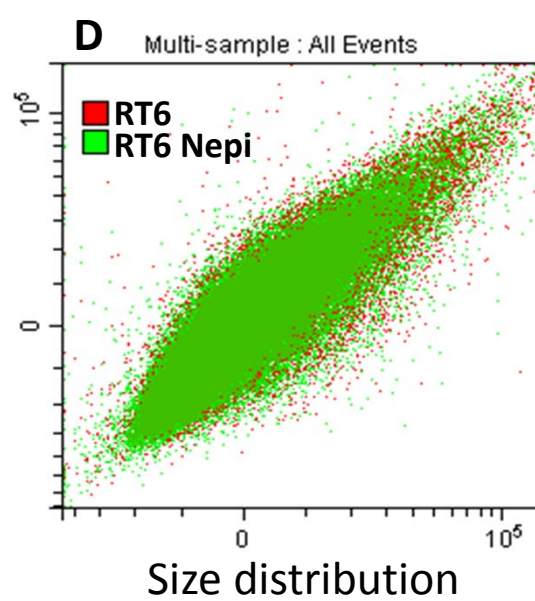

Supplement: Supplementary Data 1 — Flow cytometry graphs showing surface granularity vs. bacterial size for RT4 (A,B) and RT6 (C,D) C. acnes strains exposed to epinephrine (Epi) or norepinephrine (Nepi). [file Data_Sheet_1.PDF]
